# Immunohistochemical expression of CYP11A1, CYP11B, CYP17, and HSD3B2 in functional and nonfunctional canine adrenocortical tumors

**DOI:** 10.1111/jvim.17212

**Published:** 2024-10-10

**Authors:** Frederik Allan, Alice H. Watson, Harriet M. Syme

**Affiliations:** ^1^ Department of Veterinary Clinical Sciences, Royal Veterinary College University of London Hertfordshire United Kingdom

**Keywords:** adrenocortical neoplasia, hyperadrenocorticism, hyperaldosteronism, immunohistochemistry, steroidogenic enzyme

## Abstract

**Background:**

Functionality of human adrenal tumors is inferred by CYP11B1 (cortisol synthase) expression, CYP11B2 (aldosterone synthase) expression, or both.

**Hypothesis/Objectives:**

Nonfunctional canine adrenal tumors have low expression of steroidogenic enzymes, whereas aldosterone‐producing tumors express CYP11B, and cortisol‐producing tumors express both CYP11B and CYP17.

**Animals:**

Twenty‐two client‐owned dogs with adrenocortical tumors (ACT) (8 nonfunctional, 7‐cortisol producing, 2 aldosterone‐producing and 5 functional noncortisol producing) and 2 dogs with normal adrenal glands.

**Methods:**

Retrospective case series. Adrenal functionality was determined from clinical signs and endocrine testing. CYP11A1, CYP11B, CYP17, and HSD3B2 expression was detected by immunohistochemistry on formalin‐fixed paraffin‐embedded adrenal tissue. Protein expression was semiquantified by 2 blinded observers using H‐scoring (results reported as median [range]) and compared in nonfunctional and cortisol‐producing adrenal tumors by Mann‐Whitney *U* tests.

**Results:**

CYP11A1, CYP11B, and HSD3B2 were present within all cortical layers of normal adrenal glands, and CYP17 was expressed within the zona fasciculata and zona reticularis. Expression of CYP11A1 (191.25 [97.5‐270] vs. 175 [102.5‐295] *P* = .69), CYP11B (190 [130‐265] vs. 147.5 [95‐202.5]; *P* = .07), CYP17 (177.5 [87.5‐240] vs. 247.5 [55‐292.5]; *P* = .40), and HSD3B2 (230 [47.5‐295] vs. 277.5 [67.5‐295]; *P =* .34) were not significantly different between cortisol‐producing and nonfunctional ACT.

**Conclusions and Clinical Importance:**

Our findings suggest it is not possible to determine functionality of canine ACT by immunohistochemistry for steroidogenic enzymes. Tumor size cannot be used to infer functionality of adrenal tumors.

**Abbreviations**
17OHP: 17α‐hydroxyprogesterone
ACT: adrenocortical tumors
DAB: 3,3′‐diaminobenzidine
DOC: deoxycorticosterone
ICC: intraclass coefficient
IHC: immunohistochemistry
IMS: industrial methylated spirit
LDDS: low‐dose dexamethasone suppression test

## 1 INTRODUCTION

Unlike humans who possess 2 different enzymes for production of aldosterone (aldosterone synthase, CYP11B2) and cortisol (cortisol synthase, CYP11B1), dogs are 1 of the many species possessing a single CYP11B enzyme responsible for production of both cortisol and aldosterone. In dogs, zone‐specific expression of CYP17 in the zona fasciculata and reticularis is thought to result in cortisol production, rather than aldosterone.^1^

Aldosterone production in human functional adrenocortical tumors (ACT) can be inferred immunohistochemically by staining for CYP11B2,^2^ with further characterization according to the type of lesion (nodules, micronodules, hyperplasia),^3^ and cortisol‐producing ACT in humans have altered expression of steroidogenic enzymes compared to normal adrenal glands and nonfunctional ACT.^4^, ^5^, ^6^, ^7^ Although multiple studies in the human literature have assessed expression of steroidogenic enzymes in functional ACT, nonfunctional ACT, and normal adrenal glands,^2^, ^4^, ^6^, ^7^, ^8^, ^9^ limited studies have been performed in dogs. Investigations to date have analyzed mRNA expression of steroidogenic enzymes in dogs with cortisol‐producing ACT,^1^, ^10^, ^11^, ^12^ and no studies have assessed immunohistochemical expression of steroidogenic enzymes in dogs with different types of ACT. An improved understanding of the mechanisms of functionality of canine ACT could help guide future research toward producing improved methods for identification and treatment of affected patients and might also aid our understanding of the mechanisms of control of cortisol and aldosterone production in species possessing a single CYP11B enzyme.

The aim of our study was to assess immunohistochemical expression of steroidogenic enzymes in functional and nonfunctional canine ACT. We hypothesized that nonfunctional ACT would have lower expression of steroidogenic enzymes than cortisol‐producing ACT and that CYP17A1 expression would be high in cortisol‐producing ACT but low in aldosterone‐producing ACT.

## 2 MATERIALS AND METHODS

### 2.1 Sample collection

Medical records from the Queen Mother Hospital for Animals, Royal Veterinary College, were searched retrospectively for dogs that underwent adrenalectomy between 2002 and 2022. Histopathological records of these dogs were reviewed to identify patients with primary ACT. Inclusion criteria were adrenal histopathology confirming adrenocortical neoplasia, endocrine testing results and clinical signs consistent with a high clinical suspicion of functionality for dogs with functional adrenocortical neoplasia, and absence of clinical signs, with consistent endocrine testing results when available, in dogs with a high clinical suspicion for nonfunctional adrenocortical neoplasia. Dogs were excluded if full histopathological or clinical records were not available, if the tissue block could not be found, if a nonadrenocortical neoplastic or other pathological adrenal process was documented, if bilateral adrenalectomy was performed, or if the ACT was too large to section onto a single slide for immunohistochemistry.

Adrenals harvested from 2 dogs undergoing postmortem for nonadrenal‐disease related conditions as part of a separate histopathological study were acquired, and 1 testis was harvested from a healthy dog undergoing routine castration, with owner consent, for use as control tissues. Clinical records and postmortem findings from the control adrenal cases were not suggestive of concurrent adrenal pathology and the adrenals were deemed normal. One case died of ethylene glycol toxicity, the other of septic peritonitis after enterectomy.

Data extracted from clinical records included signalment (sex, neuter status, age), clinical signs at presentation, reason for adrenalectomy, clinical diagnosis, histopathological diagnosis, maximum recorded gross adrenal diameter, and results of endocrine testing. Endocrine testing included low‐dose dexamethasone suppression (LDDS) tests, basal and post‐ACTH stimulation cortisol concentration, urine cortisol : creatinine ratio, basal and post‐ACTH stimulation aldosterone, basal and post‐ACTH 17α‐hydroxyprogesterone (17OHP), and basal and post‐ACTH deoxycorticosterone (DOC) concentrations.

Functional ACTs were subdivided into cortisol, 17OHP, aldosterone, sex‐hormone (estradiol and progesterone), DOC, and combined 17OHP and aldosterone‐producing groups on the basis of compatible clinical signs, abnormalities on routine blood work, or both, and endocrine testing. Not all hormones were tested for in every case; testing was performed at the discretion of the attending clinician and based on the owner's wishes. Nonfunctional ACTs were classified on the basis of an absence of signs associated with endocrine hyperfunction and in some cases endocrine testing that indicated normal adrenal function.

### 2.2 Immunohistochemistry

Paraffin‐embedded blocks from cases with adrenocortical neoplasia, 2 dogs with normal adrenals collected postmortem for a separate study, and 1 testicle from a healthy dog harvested postcastration were cut into 4‐μm sections and mounted on Superfrost Plus charged slides (VWR Avantor, Pennsylvania, USA).

Sections were heated at 60°C for a minimum of 15 minutes before rehydration in a series of xylene, industrial methylated spirit (IMS) and IMS peroxidase baths. Heat‐induced epitope retrieval was performed by immersing sections in a citrate‐based solution (H‐3300 1:1000 in deionized water [Vector Laboratories, United Kingdom]) at high pressure for 10 minutes. Immunostaining was performed by an automated machine (DAKO Autostainer). Primary antibodies were diluted in emerald‐green diluent (Sigma‐Aldrich, 936B). Sections were incubated with 200 μL of either rabbit polyclonal anti‐CYP11A1 (1:1000) (Sigma‐Aldrich, HPA016436, Gillingham, United Kingdom), rabbit polyclonal anti‐CYP11B (241‐RSL, 1:500) (produced by Vohra et. al, 2020,^13^ kindly gifted by Dr. Tracey Williams), mouse monoclonal anti‐CYP17 (1:10 000) (clone 10/19‐G6‐7‐10, kindly gifted by Dr. Gomez‐Sanchez), mouse monoclonal HSD3B2 (1:1000) (produced by Gomez‐Sanchez et. al, 2017,^14^ kindly gifted by Dr. Gomez‐Sanchez) or no primary antibody for 40 minutes before rinsing. Slides stained by the SuperSensitive Polymer‐HRP IHC Detection System as per the manufacturer's instructions [QD420‐YIKEN, Biogenex] followed by a 3,3′‐diaminobenzidine (DAB) substrate to visualize bound antibody. Sections were counterstained with hematoxylin before mounting and coverslip application.

CYP11A1, CYP17, and HSD3B2 antibody concentrations were optimized on normal canine adrenal and testis sections by the described protocol, and CYP11B was optimized on normal canine adrenal sections only, considering the known locations of expression of these enzymes. Immunohistochemistry of control tissue was used to validate antibody specificity before assessment of expression of steroidogenic enzymes in ACT. All cortisol‐producing and nonfunctional tumors, 1 aldosterone‐producing tumor and 1 suspected DOC‐producing tumor were incubated with anti‐CYP11A1, anti‐CYP11B, anti‐CYP17, and anti‐HSD3B2. One aldosterone‐producing tumor and all sex‐steroid producing tumors were incubated with anti‐CYP11B and anti‐CYP17 antibodies.

Semiquantitative H‐scoring was performed on all sections as detailed in previous studies.^15^ Briefly, the H‐score was calculated by dividing the tumor into areas based on staining intensity then multiplying the area of tumor (estimated to the nearest 5%) by the staining intensity (0 = no stain, 1 = weak stain, 2 = moderate staining, 3 = strong staining), and then adding the values together, with a maximal score of 300 possible. H‐scoring was performed by 2 authors (F. Allan, A.H. Watson), blinded to the functionality of the tumors, and the mean H‐score for each section was used for further analysis.

### 2.3 Statistical analyses

Age, neuter status, clinical signs at presentation, reason for adrenalectomy, clinical diagnosis, and histopathological diagnosis are presented descriptively.

Intraclass correlation coefficient (ICC) was used to quantify interobserver measurement agreement. For ICC calculations, a 2‐way single measures mixed effect model (where all slides are measured by the same observers) for absolute agreement was selected. The agreement of the investigators performing the measurements was considered poor if the value was 0‐0.2, fair if 0.21‐0.40, moderate if 0.41‐0.6, substantial if 0.61‐0.8, and almost perfect if 0.81‐1.

Nonparametric 2‐tailed Mann‐Whitney *U* tests were performed to compare H‐scores for CYP11A1, CYP11B, CYP17, and HSD3B2 between cortisol‐producing and nonfunctional groups and to compare maximum recorded adrenal diameter between cortisol‐producing and nonfunctional groups. Results were considered significant if *P* < .05. Data are presented as median (range). No statistical comparisons were possible for 17OHP‐producing, aldosterone‐producing, sex‐steroid‐producing and DOC‐producing groups because of the rarity of these tumors; results from these groups are presented descriptively.

## 3 RESULTS

### 3.1 Cases

Overall, 107 dogs underwent adrenalectomy between 2002 and 2022. In total, 41 cases were excluded because of incomplete clinical records, histopathology, endocrine testing results, or a combination of these, 30 because of nonadrenocortical pathology, 13 because of bilateral adrenalectomy, and 1 because of mass size precluding feasibility of automated immunohistochemistry. Twenty‐two cases underwent analysis.

### 3.2 Control tissues

**FIGURE 1:**
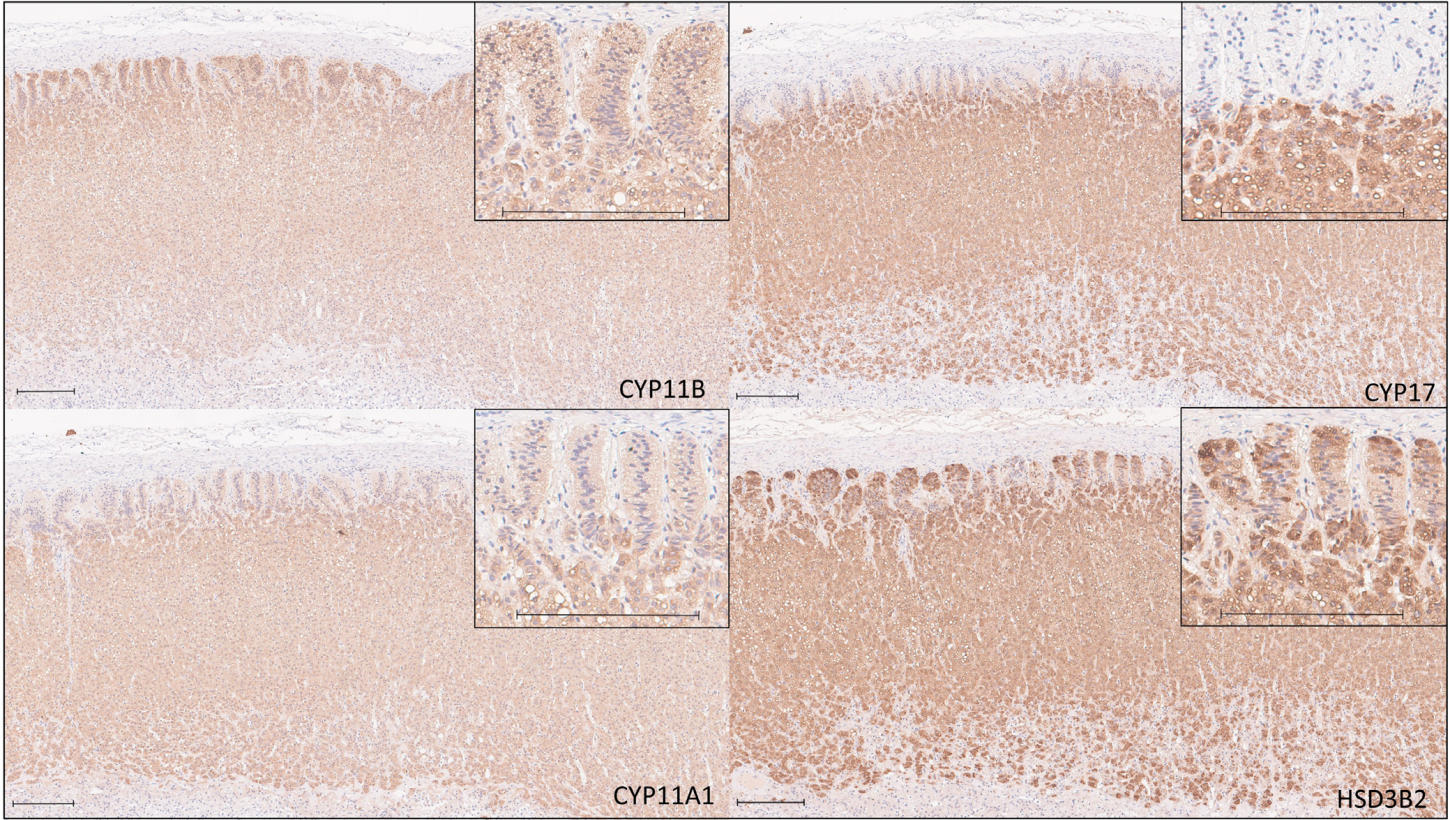
Representative example of immunohistochemical staining for CYP11B, CYP17, CYP11A1, and HSD3B2 in a normal canine adrenal gland. Bar represents 200 μm. CYP11B, CYP11A1 and HSD3B2 stain positively within all cortical layers, and CYP17 stains intensely within the zona fasciculata and reticularis. Inset are higher magnification images of the same sections, with bar representing 200 μm.

**FIGURE 2:**
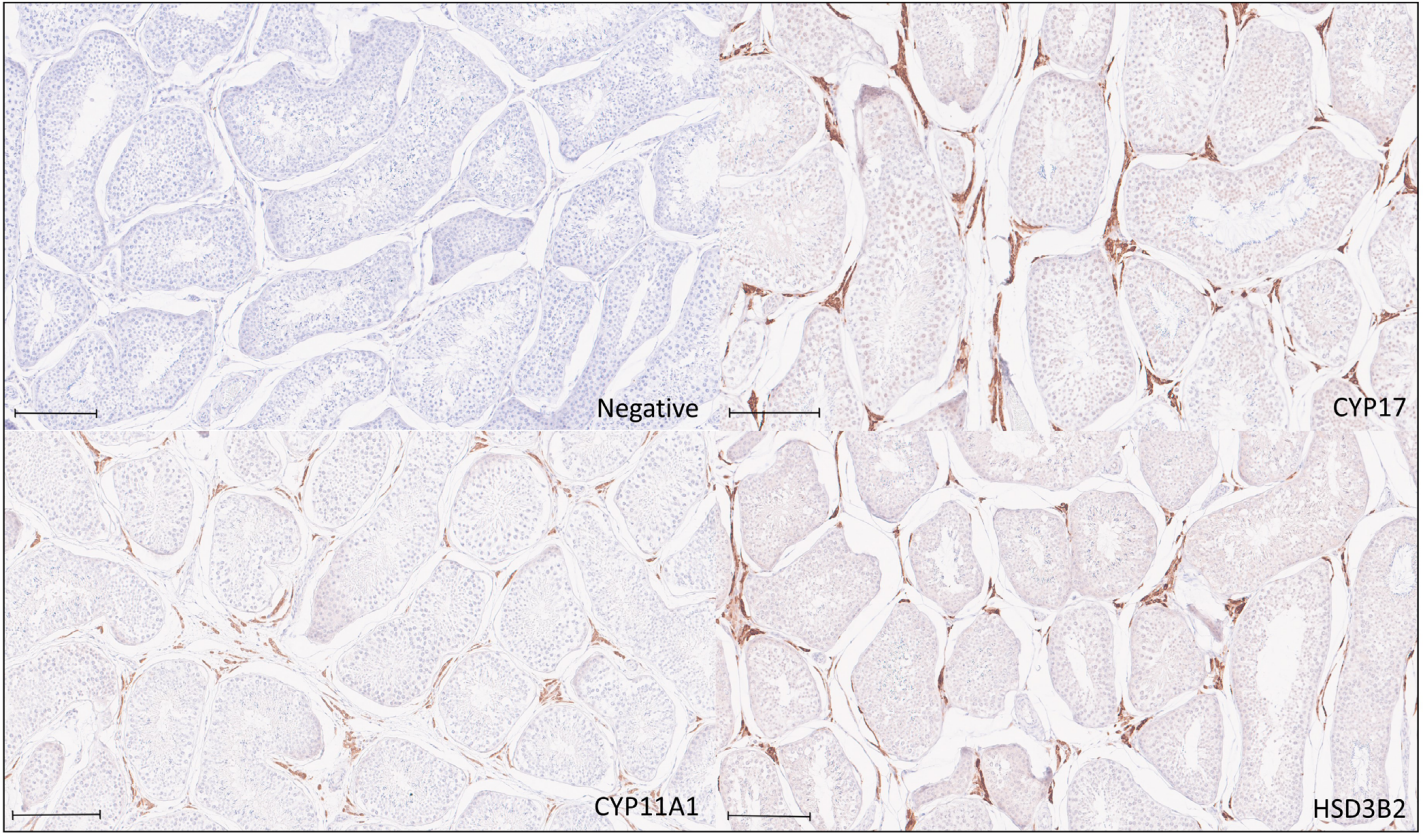
Representative example of immunohistochemical staining for CYP17, CYP11A1 and HSD3B2 in a normal canine testis, with 1 section representing no primary antibody (negative control). Leydig cells stain positively for CYP17, CYP11A1, and HSD3B2. Bar represents 200 μm.

CYP11A1, CYP11B, and HSD3B2 were present within all cortical layers of the control adrenal glands, and CYP17 was expressed within the zona fasciculata and reticularis (Figure 1). CYP11A1, CYP17, and HSD3B2 were expressed within Leydig cells of the control testis (Figure 2). Adrenal and testis slides incubated with no primary antibody did not uptake DAB stain.

### 3.3 Interobserver variability

Interobserver H‐score measurements exhibited almost perfect agreement (ICC, 0.82; 95% CI, 0.73‐0.88, *P* < .001).

## 4 ADRENOCORTICAL TUMOR CASES

### 4.1 Cortisol producing

**FIGURE 3:**
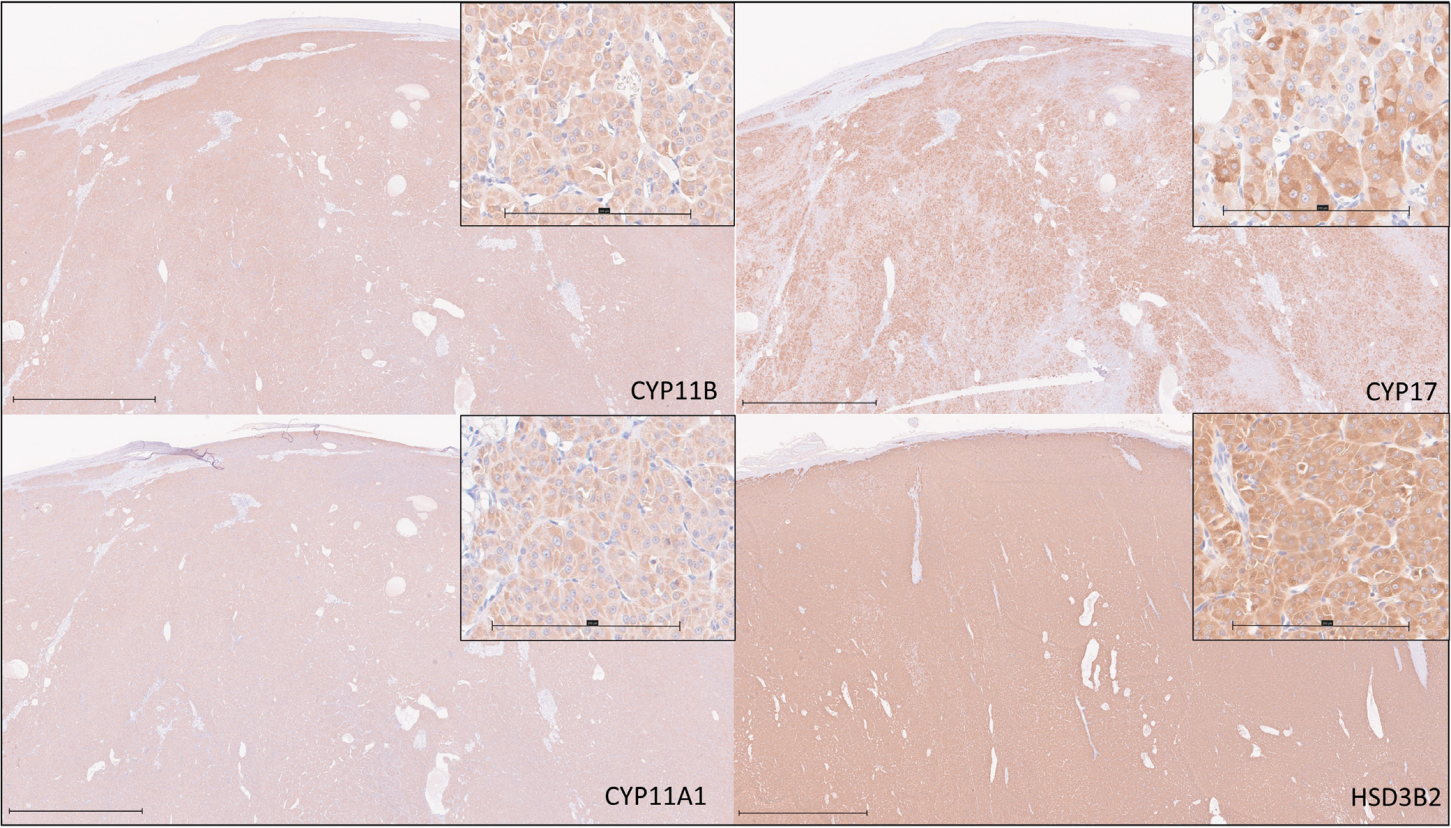
Immunohistochemical staining for CYP11B, CYP17, CYP11A1, and HSD3B2 from a section of a single cortisol‐producing adrenocortical adenoma. Bar represents 2 mm. Inset are higher magnification images of the same sections, with bar representing 200 μm.

Eight dogs with cortisol‐producing ACT were identified. Four were female‐neutered, 2 were male‐intact, and 2 were male‐neutered. Age was 10.3 [8.4‐12.6] years. Data regarding tumor diameter were available for 7 cases, median maximal adrenal diameter was 30 mm (range, 25‐46 mm). Adrenocortical carcinoma was diagnosed on histopathology in 5 cases and adrenocortical adenoma in 3. All cases had clinical signs and endocrine testing results consistent with hypercortisolism, 7 of which underwent LDDS testing, and 1 dog underwent ACTH stimulation testing. Expression of CYP11A1 (191.25 [97.5‐270]), CYP11B (190 [130‐265]), CYP17 (177.5 [87.5‐240]), and HSD3B2 (230 [47.5‐295]) was identified in all tumors (Figures 3 and 5).

### 4.2 Nonfunctional

**FIGURE 4:**
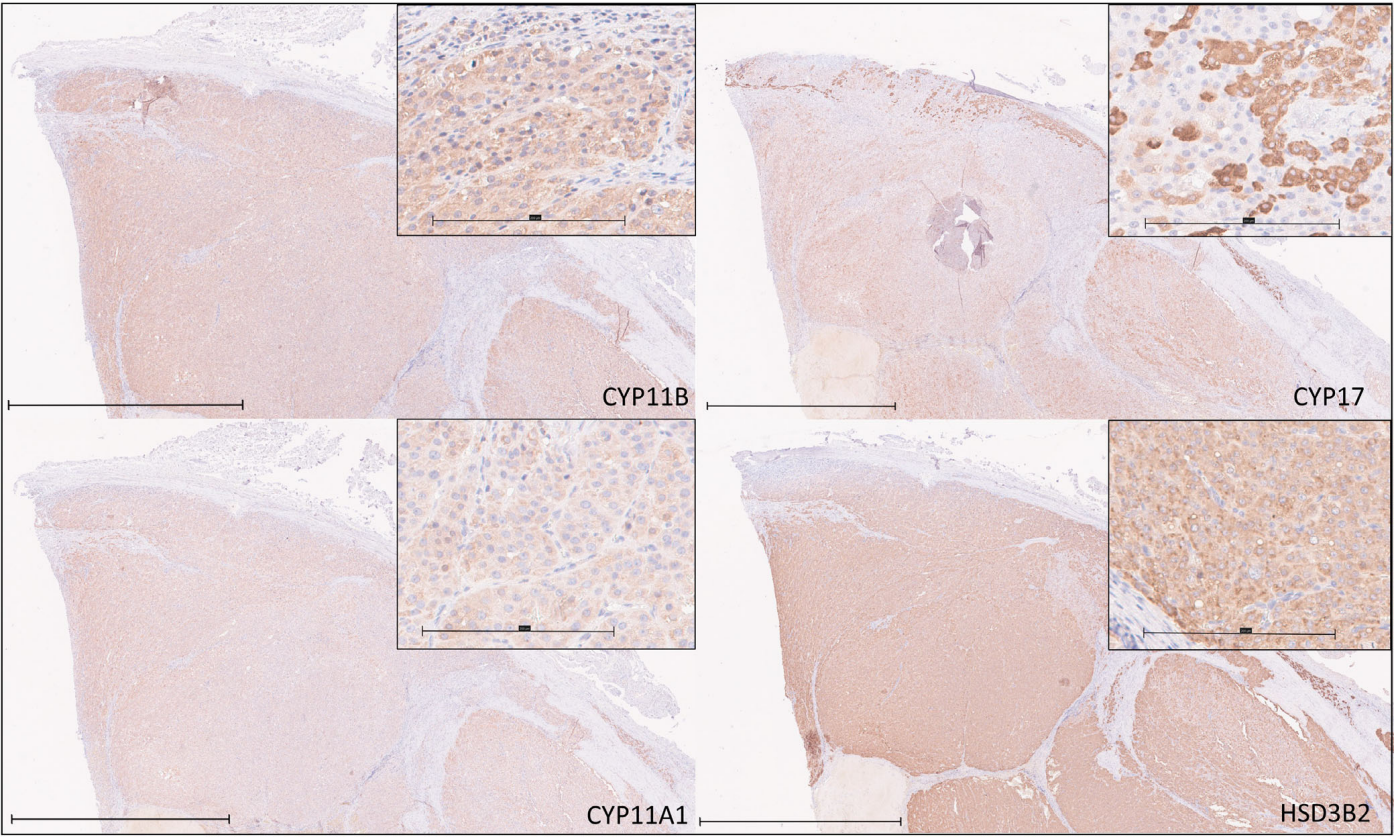
Immunohistochemical staining for CYP11B, CYP17, CYP11A1, and HSD3B2 from a section of a single nonfunctional adrenocortical adenocarcinoma. Bar represents 2 mm. Inset are higher magnification images of the same sections, with bar representing 200 μm.

**FIGURE 5:**
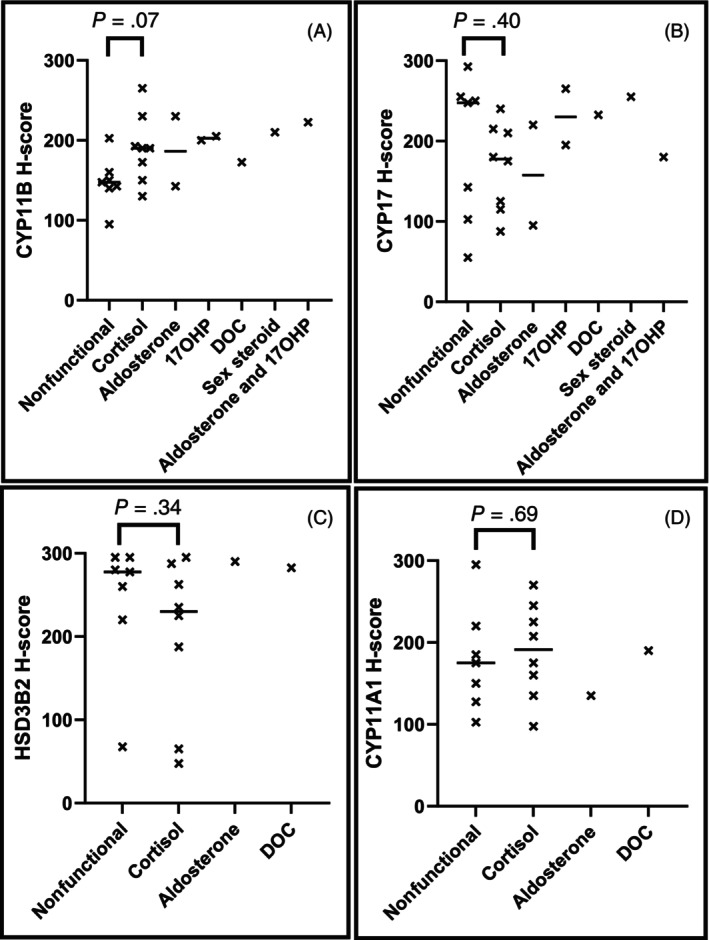
Immunohistochemical expression of CYP11B (A), CYP17 (B), HSD3B2 (C), and CYP11A1 (D) for all adrenocortical tumors in which testing was performed. The elbow bracket illustrates *P* value for H‐score distribution between cortisol‐producing and nonfunctional groups. Each cross represents individual H‐score and the horizontal lines represent median H‐score for the amount of immunohistochemical staining for each steroidogenic enzyme, where appropriate. 17OHP, 17α‐hydroxyprogesterone; DOC, deoxycorticosterone.

Seven dogs with nonfunctional ACT were identified. One was female‐intact, 2 were female‐neutered, 1 was male‐intact, and 3 were male‐neutered; median age was 10.9 [9‐11.9] years. Median maximal adrenal diameter was 48 mm (range, 19‐70 mm). Adrenocortical carcinoma was diagnosed on histopathology in 5 cases and adrenocortical adenoma in 2 cases. Four cases had adrenal masses identified while undergoing investigations for unrelated disease processes, 2 presented with ruptured adrenal masses and associated intra‐abdominal hemorrhage, and 1 case was initially suspected to be a pheochromocytoma because of episodes of weakness, hypertension and accelerated idioventricular rhythm; however, metanephrine assay results did not support this diagnosis and histology was consistent with an adrenocortical carcinoma. Four cases had endocrine testing performed (ACTH stimulation testing in 2 cases, ACTH stimulation and LDDS in 1 case and urine cortisol : creatinine ratio in 1 case) of which none were consistent with hyperfunctionality, and no case had clinical signs suggestive of functional cortical disease. Expression of CYP11A1 (175 [102.5‐295]), CYP11B (147.5 [95‐202.5]), CYP17 (247.5 [55‐292.5]), and HSD3B2 (277.5 [67.5‐295]) was scored in all tumors (Figures 4 and 5).

### 4.3 Primary hyperaldosteronism

Two dogs with primary hyperaldosteronism were identified; 1 was female neutered (4.2 years) and 1 was male neutered (10.7 years). Maximal adrenal diameter was 25 mm and 36 mm, respectively. Adrenocortical carcinoma was diagnosed in one, and adrenocortical adenoma in the other. Both cases presented with polyuria/polydipsia, muscle weakness, and hypokalemia and had increased aldosterone concentrations. Immunohistochemistry for CYP11B and CYP17 was performed in both cases, and CYP11A1 and HSD3B2 in 1 case. Expression of CYP11B (142.5 and 230) and CYP17 (95 and 220) was scored in both tumors (Figure 5). CYP11A1 and HSD3B2 were present in the single case in which IHC was performed with H‐scores of 135 and 290, respectively (Figure 5).

## 5 FUNCTIONAL NONCORTISOL‐PRODUCING

### 5.1 17α‐hydroxyprogesterone

Two dogs with 17OHP producing adrenocortical neoplasia were identified. One was female neutered (11.8 years), and the other was male neutered (11.4 years). Maximal adrenal diameters were 17 mm and 55 mm respectively. Adrenocortical carcinoma was diagnosed in both cases. Both cases presented with polyuria/polydipsia and had increased 17OHP concentrations. Immunohistochemistry for CYP11B and CYP17 was performed in both cases. CYP11B (median H‐scores 200, 205) and CYP17 (median H‐scores 195, 265) were present in both tumors (Figure 5).

### 5.2 Sex steroid

A single male neutered dog aged 10.3 years was identified with an adrenocortical carcinoma producing sex‐steroids. The dog presented with bilateral, symmetric nonpruritic alopecia, and gynecomastia, was documented to have increased progesterone and estradiol values and had LDDS testing not consistent with hypercortisolism. Maximal adrenal diameter was 50 mm. CYP11B and CYP17 were expressed with H‐scores of 210 and 255, respectively (Figure 5).

### 5.3 17OHP and aldosterone

A single female‐neutered dog aged 11.3 years with an adrenocortical carcinoma producing 17OHP and aldosterone was identified. The dog presented with hypokalemia, hypertension, polyuria and polydipsia, vulval discharge, and a stump pyometra, with increased 17OHP values pre‐ and post‐ACTH, and increased basal aldosterone. ACTH stimulation testing was not consistent with a cortisol‐secreting ACT. Maximal adrenal diameter was 40 mm. CYP11B and CYP17 were both expressed with H‐scores of 222.5 and 180, respectively (Figure 5).

### 5.4 Deoxycorticosterone

A single female neutered dog aged 9.2 years with an adrenocortical carcinoma producing DOC was identified. The dog presented with polyuria and polydipsia, moderate persistent hypokalemia, hypertension, and neck pain. ACTH stimulation, LDDS, and aldosterone assays were not consistent with hypercortisolism or hyperaldosteronism (aldosterone concentration was actually suppressed), and DOC concentration was increased consistent with a DOC‐producing mass. Maximal adrenal size was 2.6 × 3.5 cm on ultrasound (also had computed tomography [CT]). Clinical signs resolved after adrenalectomy. CYP11A1 (190), CYP11B (172.5), CYP17 (232.5), and HSD3B2 (282.5) were expressed (Figure 5).

### 5.5 Cortisol‐secreting versus nonfunctional adrenocortical neoplasms

No significant differences in expression of CYP11A1 (*P* = .69), CYP11B (*P* = .07), CYP17 (*P* = .40) or HSD3B2 (*P* = .34) were found between cortisol‐secreting and nonfunctional ACT (Figure 5). No significant difference in maximal adrenal size was detected between cortisol‐secreting and nonfunctional ACT (*P* = .21).

## 6 DISCUSSION

Increased expression of steroidogenic enzymes has been identified in human functional ACT compared to normal adrenal glands by RNA microarray analysis^7^ and nonfunctioning ACT.^5^ These findings have not been mirrored by canine studies evaluating mRNA expression of steroidogenic enzymes in cortisol‐secreting ACT compared to normal adrenals^10^ or in the present study assessing immunohistochemical steroidogenic enzyme expression in canine functional ACT compared with nonfunctional ACT. There are several possible explanations for the contrasting findings of studies investigating the relationships between steroidogenic enzyme expression and functionality of ACT in humans and dogs so far. This could be attributable to differences in methodologies, criteria used to classify functionality, and volumes of specimens analyzed, or could indicate that the pathogenesis underlying functionality of ACT in dogs differs to that in humans. Our findings suggest we cannot retrospectively determine functionality of canine ACT using immunohistochemistry.

In primates and rodents, production of aldosterone is reliant on expression of CYP11B2 (aldosterone synthase), and production of cortisol (or corticosterone in the case of rodents) relies predominantly on expression of CYP11B1 (cortisol synthase),^16^ whereas in dogs, cats, birds, sheep and pigs a single CYP11B is responsible for production of both aldosterone and cortisol.^16^ In healthy dogs, zone‐specific production of aldosterone and cortisol is thought to be related to higher expression of CYP17 in the zona fasciculata than the zona glomerulosa, resulting in cortisol synthesis in the zona fasciculata and aldosterone synthesis in the zona glomerulosa.^1^ Immunohistochemical expression of CYP11B in normal canine adrenals was evident in all 3 layers of the adrenal cortex in our study, with more intense immunohistochemical expression of CYP17 in the zona fasciculata and zona reticularis than the zona glomerulosa, similar to the immunohistochemical staining pattern of CYP17 documented in the normal canine adrenal cortex in a previous study.^1^ The pattern of immunohistochemical expression of CYP11B, CYP17, CYP11A1, and HSD3B2 in normal canine adrenal glands, and in Leydig cells, in our study suggests that the antibodies used were specific to the proteins of interest and are valid for assessment of steroidogenic enzyme expression in dogs.

Assessment of immunohistochemical expression of CYP11B2 is the recommended method for identifying regions of aldosterone production in human adrenal glands with primary hyperaldosteronism.^2^ In dogs' adrenal glands, if steroidogenic enzyme expression was directly implicated in functionality, then an absence or reduced expression of CYP17 with high expression of CYP11B would be expected in aldosterone‐producing ACT compared with cortisol‐producing ACT. This is because CYP17 is necessary for cortisol production, and its absence in the zona glomerulosa suggests expression of CYP17 could direct steroid precursors away from aldosterone production. Whilst no statistical comparison was possible because of the rarity of aldosterone‐producing adrenal tumors in dogs, expression of CYP17 was similar in both primary hyperaldosteronism cases to cortisol‐producing cases and nonfunctional cases. This also suggests that steroidogenic enzyme expression is not the main driver of functionality of canine ACT.

Our study found no significant difference in size between cortisol‐producing and nonfunctional tumors, indicating that the volume of tumor tissue was not lower in the tumors that were classified as nonfunctional. Retrospective assessment of adrenal tumor size is challenging, however, and whilst studies commonly use maximal tumor axis or maximal tumor diameter as an indicator of tumor size,^17^, ^18^ this could misestimate true tumor volume, which is a possibility in the present study.

A limitation of our study was that dogs in the nonfunctional group did not have extended steroid panels performed, and it cannot be ruled out that production of an unmeasured or nonmeasurable steroid was occurring in these cases without resulting in clinical signs or resulting in clinical signs that were simply not recognized. ACTH stimulation testing has poor sensitivity for adrenal‐dependent hyperadrenocorticism, and therefore cases assigned as nonfunctional based on ACTH stimulation testing and a reported absence of clinical signs could have been misclassified. The absence of any significant difference in steroidogenic enzyme expression between cortisol‐producing ACT and nonfunctional ACT in our study might suggest that perceived nonfunctional ACT in dogs possess steroidogenic activity. Similarly, expression of steroidogenic enzymes have been documented in “nonfunctional” human adrenal tumors^4^, ^6^, ^9^ with investigators suggesting that these tumors could have steroidogenic activity considering their similar enzyme expression to normal control adrenals, despite a lack of clinical signs of functionality.

Three cases categorized as functional in our study were found to have increased 17OHP values, 1 case had concurrent hyperaldosteronism. Interestingly, immunohistochemical expression of CYP11B and CYP17 was present in all 3 cases, which is consistent with a theoretical capacity to produce cortisol, aldosterone and 17OHP. As previously mentioned, the poor sensitivity of ACTH stimulation testing in adrenal‐dependent hyperadrenocorticism,^19^ could have led to a failure to detect cortisol excess in these cases. Both the use of 17OHP for diagnosis of hyperadrenocorticism and the relationship of increased 17OHP with clinical signs in dogs are debated in the veterinary literature.^20^ Excessive 17OHP alone is not expected to cause clinical signs in humans.^8^, ^21^ As 17OHP is not routinely measured in the absence of clinical signs of cortisol‐excess, the importance of increased 17OHP in the few symptomatic cases in which it was measured is unclear. It is possible that 17OHP acts as a measurable precursor to another nonmeasured functional steroid in these clinical cases.

Although our study is the first to investigate immunohistochemical expression of steroidogenic enzymes in functional and nonfunctional canine ACT, there are several limitations. Firstly, the retrospective nature of our study meant that variations exist in case management, diagnostics performed, and steroid panels measured, which might have impacted determination of functionality. Immunohistochemical labeling is impacted by multiple preanalytical variables including time to fixation, time in fixative and storage of slide‐mounted sections,^22^ all of which could have impacted the results of the present study. However, given these were clinical specimens, it is likely the tumors would have been immediately fixed in formalin and processed into formalin‐fixed paraffin embedded (FFPE) samples within 24‐72 hours of collection. Multiple scoring systems have been proposed for assessment of immunohistochemical labeling, with H‐scoring used in the present study to facilitate practicality of assessing large numbers of sections. This semiquantitative approach comes with a degree of subjectivity when compared to more quantitative methods, however the near perfect interobserver agreement in our study suggests effective assessment. Although automated systems might be able to analyze slides more efficiently, the sections in our study were heterogeneous tumors and some sections had both tumor and normal adrenal coexisting on the same slide, which could lead to analytic error when automated. The heterogeneity of tumors poses another limitation of the present study, as examined sections might not have been wholly representative of the tumor assessed. It would be interesting to assess adrenal tissue adjacent to tumors for relative size of the zona fasciculata and zona reticularis compared with the zona glomerulosa, as presence of zona fasciculata atrophy could be supportive of cortisol excess. This analysis was not possible in the present study because of the small numbers of cases with normal adrenal tissue coexisting on the same slide and the tumor disrupting the normal adrenal architecture. Assessment of functionality of canine ACT is challenging in the clinical setting, with variable sensitivity and specificity of frequently used endocrine testing. The possibility of incorrect determination of functionality cannot be discounted, which would negatively impact the results of our study.

In conclusion, immunohistochemistry for the steroidogenic enzymes CYP11B, CYP17, CYP11A1 and HSD3B2 was not useful for the retrospective determination of functionality of ACT in this cohort of dogs. A secondary finding of our study was that adrenocortical tumor size did not differ between functional and nonfunctional cases, indicating that lack of functionality was not because of decreased volume of tumor tissue.

## CONFLICT OF INTEREST DECLARATION

Authors declare no conflict of interest.

## OFF‐LABEL ANTIMICROBIAL DECLARATION

Authors declare no off‐label use of antimicrobials.

## INSTITUTIONAL ANIMAL CARE AND USE COMMITTEE (IACUC) OR OTHER APPROVAL DECLARATION

Authors declare no IACUC or other approval was needed.

## HUMAN ETHICS APPROVAL DECLARATION

Authors declare human ethics approval was not needed for this study.

## Supporting information


**Data S1.** Supporting Information.
