# Supplementary material for: Immunohistochemical expression of CYP11A1, CYP11B, CYP17, and HSD3B2 in functional and nonfunctional canine adrenocortical tumors
Source: J Vet Intern Med. 2024 Oct 10;38(6):3070–8. doi: 10.1111/jvim.17212 (PMC11586580; doi:10.1111/jvim.17212)
Supplement: Supplementary file 1 — Data S1. Supporting Information. [file JVIM-38-3070-s001.docx]

**Supplemental information: endocrine testing results and clinical signs.**

| **ID** | **Functionality** | **Clinical signs** | **Basal cortisol nmol/l (RI <250)** | **3h post-dexamethasone cortisol nmol/l** | **8h post-dexamethasone cortisol nmol/l (RI <40)** | **Post-ACTH cortisol nmol/l (RI <500)** | **Basal aldosterone pmol/l** | **Post-ACTH aldosterone pmol/l** |
| --- | --- | --- | --- | --- | --- | --- | --- | --- |
| 1 | Aldosterone | PUPD, hypokalaemia, stiff hindlimbs, weakness | n/a | n/a | n/a | n/a | >3000 (RI <96) | n/a |
| 2 | Aldosterone | PUPD | 87.9 | n/a | n/a | 260 | 685 (RI <393) | 2013 (RI 82-859) |
| 3 | Cortisol | PUPD, polyphagia, reduced HL proprioception | 105 | 97.2 | 114 | n/a | n/a | n/a |
| 4 | Cortisol | PUPD, panting, hair-thinning, hyperpigmentation, rat-tail | 107 | 114 | 92.2 | n/a | n/a | n/a |
| 5 | Cortisol | PUPD, polyphagia, alopecia cervical spine | 76.5 | 57.7 | 53.8 | n/a | n/a | n/a |
| 6 | Cortisol | PUPD, polyphagia | *reported consistent with hyperadrenocorticism on referral report, exact values not available | *reported consistent with hyperadrenocorticism on referral report, exact values not available | *reported consistent with hyperadrenocorticism on referral report, exact values not available | *reported consistent with hyperadrenocorticism on referral report, exact values not available | n/a | n/a |
| 7 | Cortisol | PUPD, polyphagia, abdominal distension, panting, weight gain, exercise intolerance | 81.4 | 57.7 | 60.4 | n/a | n/a | n/a |
| 8 | Cortisol | Polydipsia, potbelly appearance, hematuria, lethargy | 73 | n/a | n/a | >2000 | n/a | n/a |
| 9 | Cortisol | PUPD, polyphagia, hypertension, nocturia, lethargy, facial alopecia | 65 | 61 | 87 | n/a | n/a | n/a |
| 10 | Cortisol | PUPD, potbelly appearance | 79.7 | 90.5 | 89.9 | n/a | n/a | n/a |

Table 1: A table describing clinical signs and endocrine testing results of aldosterone-producing and cortisol-producing adrenocortical tumor cases.

| **ID** | **Functionality** | **Clinical signs** | **Basal cortisol** | **3h post-dexamethasone cortisol** | **8h post-dexamethasone cortisol** | **Post-ACTH cortisol** | **Basal aldosterone pmol/l** | **Post-ACTH aldosterone pmol/l** | **Other** |
| --- | --- | --- | --- | --- | --- | --- | --- | --- | --- |
| 11 | DOC-producing | Hypokalaemia, and neck pain (resolved post-adrenalectomy), PUPD, hypertension, hyposthenuria. | <27.6 (pre-ACTH); 72.9 (pre-LDDS) | 57.1 | 50 | 111 | 20 (RI <960 – different assay to other cases) | 20 | n/a |
| 12 | Non-functional | Collapse, lethargy, abdominal pain, liver mass, incidental enlarged right adrenal removed at time of liver lobectomy. **Unrelated disease process.** | Referral report states ACTH stimulation test and LDDS reported normal – exact values unavailable | Referral report states ACTH stimulation test and LDDS reported normal – exact values unavailable | Referral report states ACTH stimulation test and LDDS reported normal – exact values unavailable | Referral report states ACTH stimulation test and LDDS reported normal – exact values unavailable | n/a | n/a | n/a |
| 13 | Non-functional | Lameness, intermittent diarrhoea, chronic cough. Large adrenal mass identified on AUS. **Unrelated disease process.** | n/a | n/a | n/a | n/a | n/a | n/a | UCCR 24 (RI <30x10^6^ ) |
| 14 | Non-functional | Ruptured adrenal mass with retroperitoneal bleeding | 121 | n/a | n/a | 220 | n/a | n/a | n/a |
| 15 | Non-functional | Adrenal mass identified on ultrasound >1y prior to presentation during investigations for pancreatitis and haemorrhagic enteritis (**unrelated disease process**). No subsequent clinical signs but mass removed as growing on repeat monitoring. | Not performed, normal contralateral adrenal and no clinical signs. | Not performed, normal contralateral adrenal and no clinical signs. | Not performed, normal contralateral adrenal and no clinical signs. | Not performed, normal contralateral adrenal and no clinical signs. |  |  |  |
| 16 | Non-functional | Vomiting, diarrhoea, poorly controlled DM, normal contralateral adrenal. **Unrelated disease process.** | 41.7 | n/a | n/a | 428 |  |  |  |
| 17 | Non-functional | Acute weakness, tachycardia, hypertension, AIVR, normal contralateral adrenal. Suspected pheochromocytoma at time of admission and normal contralateral adrenal gland. | n/a | n/a | n/a | n/a |  |  | Plasma metanephrine 0.4mol/l (RI 0.28-3.88); plasma normetanephrine 1.87nmol/l (RI 0.95-5.38) |
| 18 | Non-functional | Ruptured bleeding mass, haemoabdomen, lethargy, acute abdominal discomfort | n/a | n/a | n/a | n/a |  |  |  |
| 19 | Aldosterone and 17OHP | Vulval discharge, stump pyometra, hypokalaemia, hypertension, PUPD, anorexia, incontinence | 74.8 | n/a | n/a | 202 | 487 (RI <393) | n/a | Pre-ACTH 17OHP 4.1nmol/l (RI <3), post-ACTH 17OHP 14.1nmol/l (RI 3-8) |
| 20 | 17-OHP | Weight loss, PUPD, chronic small bowel diarrhoea | Pre-LDDS 39.2; pre-ACTH 49.4 | 31.5 | <27.6 | 74.5 |  |  | Pre-ACTH 17OHP 1.6nmol/l (RI <3), post-ACTH 17OHP 48.0nmol/l (RI 3-8) |
| 21 | 17-OHP | PUPD | 63.4 | n/a | n/a | 68.4 | n/a | n/a | Pre-ACTH 17OHP 32nmol/l (RI <3), post-ACTH 17OHP 77nmol/l (RI 3-8) |
| 22 | Progesterone and oestradiol | Bilateral symmetric non-pruritic alopecia, gynacomastia | 284 | <27.6 | <27.6 | n/a | n/a |  | Progesterone 18nmol/l (RI <3), oestradiol 68pmol/l (RI <10), androstenodione 0.7nmol/l (RI <0.8) |

Table 2: A table describing clinical signs and endocrine testing results of DOC-producing, non-functional, and sex-steroid producing adrenocortical tumor cases. Abbreviations – AIVR, accelerated idioventricular rhythm; DM, diabetes mellitus; PUPD, polyuria/polydipsia; 17OHP, 17-hydroxyprogesterone.
